# Corepressor diversification by alternative mRNA splicing is species specific

**DOI:** 10.1186/s12862-016-0781-2

**Published:** 2016-10-19

**Authors:** Martin L. Privalsky, Chelsea A. Snyder, Michael L. Goodson

**Affiliations:** Department of Microbiology and Molecular Genetics, College of Biological Sciences, University of California at Davis, One Shields Avenue, Davis, CA 95616 USA

**Keywords:** Corepressor, NCoR, SMRT, mRNA splicing, Evolution

## Abstract

**Background:**

SMRT and NCoR are corepressor paralogs that help mediate transcriptional repression by a variety of transcription factors, including the nuclear hormone receptors. The functions of both corepressors are extensively diversified in mice by alternative mRNA splicing, generating a series of protein variants that differ in different tissues and that exert different, even diametrically opposite, biochemical and biological effects from one another.

**Results:**

We report here that the alternative splicing previously reported for SMRT appears to be a relatively recent evolutionary phenomenon, with only one of these previously identified sites utilized in a teleost fish and a limited additional number of the additional known sites utilized in a bird, reptile, and marsupial. In contrast, extensive SMRT alternative splicing at these sites was detected among the placental mammals. The alternative splicing of NCoR previously identified in mice (and shown to regulate lipid and carbohydrate metabolism) is likely to have arisen separately and after that of SMRT, and includes an example of convergent evolution.

**Conclusions:**

We propose that the functions of both SMRT and NCoR have been diversified by alternative splicing during evolution to allow customization for different purposes in different tissues and different species.

**Electronic supplementary material:**

The online version of this article (doi:10.1186/s12862-016-0781-2) contains supplementary material, which is available to authorized users.

## Background

The nuclear hormone receptors are ligand-modulated transcription factors involved in the regulation of many developmental and homeostatic processes (1–8). The transcriptional properties of these receptors are mediated through their recruitment and release of auxiliary proteins, denoted corepressors and coactivators [1–8]. SMRT and NCoR are important corepressors for the nuclear hormone receptors and for many additional, non-receptor transcription factors [3, 9–15]. Both SMRT and NCoR function as bridging proteins, possessing both specific domains that recruit them to their transcription factor partners and separate regions that bind additional components of a larger corepressor complex; the complex inhibits transcription by modifying the chromatin template and/or by interfering with components of the transcriptional machinery [3, 16].

SMRT and NCoR are present as two distinct loci in vertebrate genomes. In contrast, only single SMRT/NCoR-like loci have been detected in the genomes of other deuterostomes, including the non-vertebrate chordata and echinoderms [17]. This supports the concept that NCoR and SMRT are paralogs that arose from an ancestral gene duplication and divergence. More weakly related loci (e.g., SMRTER and GEI-8) are found in yet more divergent phyla such as the arthropoda and nematoda [18, 19] although these bear only isolated blocks of sequence relatedness and lack many other structural features found in the chordate NCoR/SMRT (such as the “CoRNR box” motifs/Receptor Interaction Domains (RIDs) responsible for the interaction of the vertebrate corepressors with their nuclear receptor partners).

**Fig. 1:**
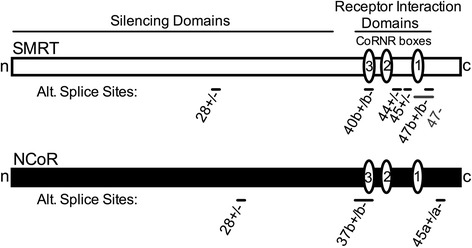
Schematic of alternative splice sites in SMRT and NCoR. The longest known corepressor alternative splice variants are depicted as horizontal rectangles. Alternative splice locations are shown as bars and exon numbers are indicated as in RefSeq. Interaction domains for additional components of the corepressor complex (“silencing domains”) and for nuclear hormone receptors (“receptor interaction domains” containing the CoRNR box motifs, represented as *ovals*) are shown

Both SMRT and NCoR in mice and humans are expressed by extensive alternative mRNA splicing to yield a series of protein variants that are expressed at different relative abundances in different tissues, possess different RIDS, have different affinities for their partner transcription factors, and exert divergent, even opposing, biological roles [17, 20–29] (Fig. 1). Alternative splicing of SMRT, but not NCoR, has also been reported at many of the same locations in *Xenopus* as in mammals, although differences in the proportions of each splice derivative were observed between the different taxa [25, 30]. Given the crucial role of alternative splicing in regulating the biological actions of SMRT and NCoR, we sought to better understand when these important regulatory modifications of corepressor function appeared during evolution. Here we report that only a limited form of this alternative splicing of SMRT can be found in a teleost fish, whereas there is increasing elaboration of this alternative splicing in a reptile, bird, amphibian, and marsupial, with the most extensive alternative splicing found in placental mammals. In contrast, extensive alternative splicing of NCoR at the locations identified in mice was limited to the placental mammals. Although several of these alternative splicing events generate variants of SMRT versus NCoR that possess similar overall molecular architectures, the underlying splicing events occur at non-homologous sites in these two corepressor loci. This suggests that these distinct alternative splicing events most likely arose in these two paralogs after their genomic duplication and that the apparent similarities are a reflection of a convergent evolution in response to common selective pressures. Notably, novel additional alternative splice sites not identified in the mouse corepressors were found in *Danio* and in *Drosophila*. We propose that alternative RNA splicing of these corepressors allows customization of SMRT and NCoR function for different purposes in different tissues at different times in different taxa.

## Methods

### Isolation of RNA from tissues and organs

All animals were maintained and euthanized using University of California at Davis authorized protocols and with the written approval of the University of California at Davis Animal Use committee; samples of human materials were previously collected and encoded to ensure anonymity, allowing them to be defined as exempt from regulation by the U. C. Davis Internal Review Board. Livers, kidneys, skeletal muscle, whole brain, cerebral cortex, cerebellum, brain stem, S1 cortex, hippocampus, and magnocellular preoptic nuclei were obtained by manual dissection. Biological samples were typically stored in RNAlater solution (Life Technologies, Grand Island, NY) at −20 or −80 °C before RNA extraction. Total RNA was isolated from approximately 35 mgs wet weight of each organ using a RNAeasy mini-kit (Qiagen, Hilden, Germany) and manufacturer’s directions [24, 29].

### cDNA synthesis and reverse transcriptase PCR quantitation

Approximately 1 μg of total RNA from each sample was converted into cDNA using a QuantiTect Reverse Transcription Kit (Qiagen, Hilden, Germany) employing the manufacturer’s directions and including a DNA-wipeout pre-step to avoid genomic contamination [24, 29]. The products were then typically diluted with RNase-free water to 120 μl, with 2 μl of which used per PCR reaction. The PCR reactions were performed in a total of 20 μl volume per reaction using the GoTaq enzyme, buffers (Promega, Madison WI), and splice-specific primer pairs designed to flank each known alternative splice-site. The multiple alternatively spliced locations within a corepressor mRNA were therefore analyzed by using a panel of primer pairs, each specific for an alternatively spliced location [24, 26, 29] (Additional file 1: Table S1). PCR was performed at 94 °C for 2 mins., followed by 30 cycles of 30 s. at 94 °C, 30 s. at 53 °C, 60 s. at 72 °C, and a final 5 min. extension at 72 °C prior to temporary storage at −20 °C.

The PCR products for each primer pair were then resolved by electrophoresis in a 2 % agarose gel in 1X TAE buffer, and the DNA was stained with ethidium bromide and was quantified by use of a digital camera and AlphaEase software (version 3.1.2) [24, 26, 29]. The abundance of each alternatively spliced corepressor isoform was calculated as a percentage of the sum of all the alternatively spliced isoforms produced at that alternative splice location (the primers were designed such that each isoform yielded a distinct-sized PCR product) [24, 26, 29]. Although this method does not quantitate absolute levels of each RNA, it does permit the relative ratio of the splice variants produced at any given alternative splice site to be accurately measured: the technique is internally controlled for primer/PCR reaction efficiency, is insensitive to possible non-uniformity in lane loading, electrophoresis, staining, or destaining, and its reproducibility has been confirmed in practice by the low standard deviations observed when this approach was repeated at distinct times or by different researchers [24, 26, 29]. Quantitative RT-PCR could not be used in this context because many corepressor variants differ from others only due to lack of specific exons, prohibiting the design of oligonucleotide primer pairs unique to these variants [24, 26, 29].

Each sample was analyzed by PCR a minimum of 3 times and the average and standard error calculated. For *Danio*, *Gallus*, *Mus*, and *Homo* (representing a wide range of taxonomy) we were able to analyze samples from three or more individuals from each species; the variation between these individuals was small and essentially equal to the variation when a sample from one individual was analyzed multiple times (the corresponding figures include this data from multiple individuals). It was more difficult to obtain multiple individuals of *Xenopus*, *Monodelphis*, or *Trachemys* due to practical or ethical reasons; here too, however, there were only minimal variations when different individuals at distinct developmental stages or different aliquots of the same tissue were analyzed individually (please see Results). We also emphasize that our conclusions are based on the qualitative presence or absence of a given splice form, rather than on any modest quantitative differences in splice abundance.

## Results

A limited form of the known alternative splicing of SMRT, but not of NCoR, could be detected early in phylogeny in the teleost zebrafish (*Danio rerio*). We began with liver as a model organ; the livers of mice and *Xenopus* express a representation of all the alternative spliced variants of both SMRT and NCoR reported for these species [21]. Notably our analysis was designed to detect all of the known splice sites that had been previously identified in *Xenopus*, mice, or humans [25].

**Fig. 2:**
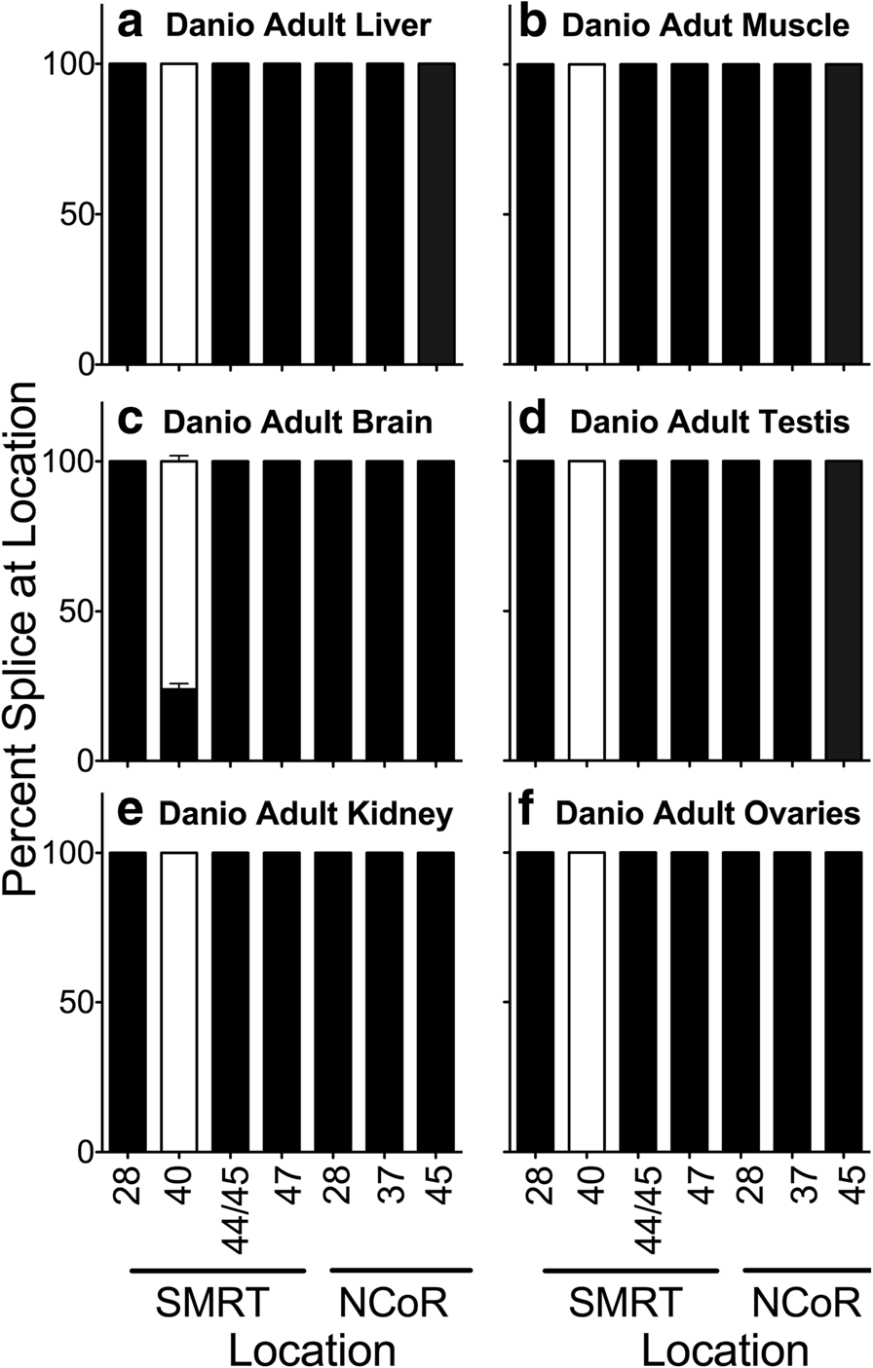
Alternative splicing in adult *Danio rerio*. The organs indicated were isolated, RNA was extracted, and the alternative splicing at the indicated locations was analyzed by use of suitable primers and RT-PCR as in Methods. The percentage of each alternatively spliced corepressor variant produced at each location was quantified by scanning and is presented as stacked bars adding to 100 %. Mean and standard deviation are shown (*n* = 5) for each. *Black* filled bars represent the longest splice variant at each location and open fill represents the shortest splice variant at each location. Panels represent Danio adult liver (**a**), Danio adult muscle (**b**), Dano adult brain (**c**), Danio adult testis (**d**), Danio adult kidney (**e**), and Danio adult ovaries (**f**)

No alternative RNA splicing of either SMRT or NCoR was detected in adult *Danio* liver at any of the sites previously found to be alternatively spliced in mice and/or *Xenopus* (Fig. 2a and Additional file 1: Figure S1A). We also tested additional *Danio* organs, including muscle, brain, testis, kidneys, and ovaries (Fig. 2b-f and Additional file 1: Figure S1B). Notably only the brain-derived material displayed alternative corepressor splicing at any of these sites: a very low level of expression of the 40b + exon of SMRT in addition to the much higher expression of the SMRT exon 40b- splice variant. The SMRT exon 40b + variant is also enriched in the brains of mouse and *Xenopus* [23, 25], although at much higher levels in these species than was observed in *Danio*; conversely the SMRT exon 40b + splice is prominent in mouse testis but this was not observed in *Danio* testis (Fig. 2) [23]. The SMRT 40b + exon splice inserts a third CoRNR box/RID domain and thus is distinct from the two RID variant encoded by the SMRT exon 40b- splice; the later was identified first and is often viewed as the prototypic form of SMRT [31–38] (Fig. 1). The SMRT 40b + and 40b- splice variants display very different preferences for different transcription factors, most clearly seen as a highly divergent ability to interact with the thyroid hormone receptor (TR) and with the peroxisome-proliferator activating receptor (PPAR)-γ, both of which play important roles in overall metabolism [22, 23].

**Fig. 3:**
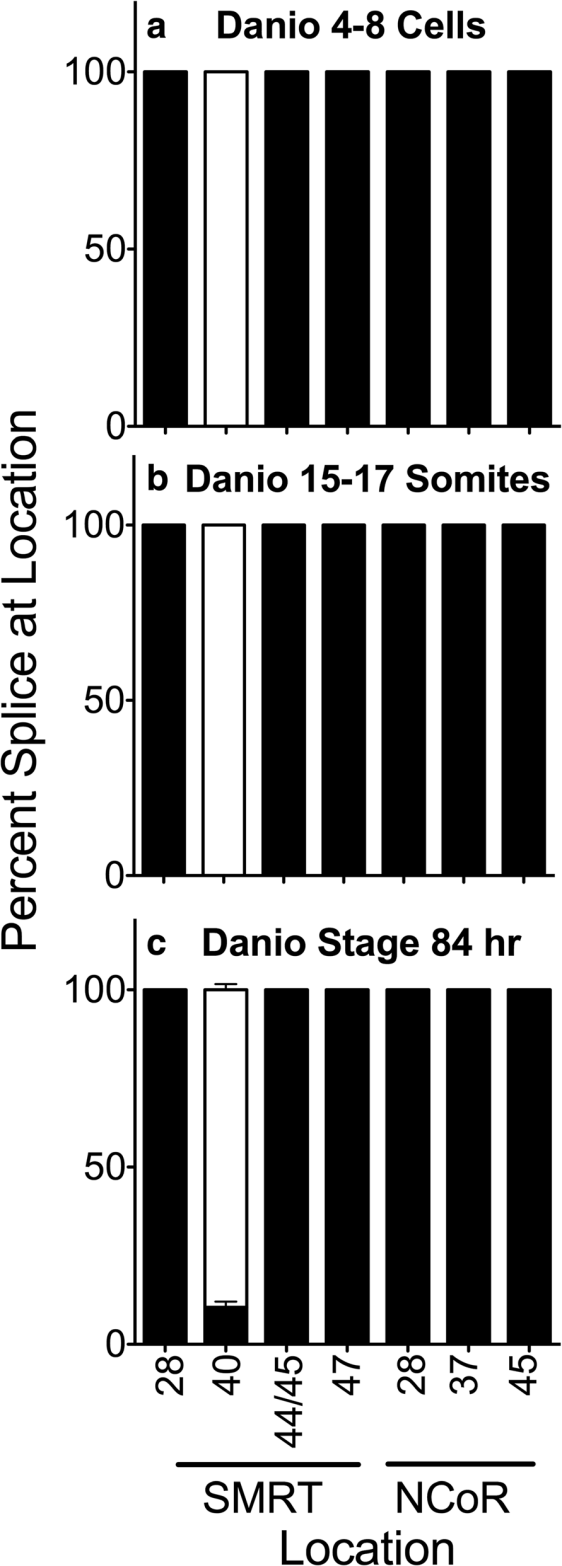
Alternative splicing during *Danio rerio* development. Entire individuals were isolated at the stages indicated, RNA was extracted, and the alternative splicing at the indicated locations was analyzed and is presented graphically as in Fig. 2. Mean and standard deviation are shown (*n* = 3) for each. Panels represent Danio 4-8 cell stage animals (**a**), Danio 15-17 somite stage animals (**b**) and Danio stage 84 hour animals (**c**)

We next asked if we could detect SMRT exon 40b + or any of other of the known corepressor splice variants in *Danio* at other developmental stages (Fig. 3). No evidence of any of these alternative corepressor variants were found in whole specimens at the 4–8 cell or 15–17 somite stages (Fig. 3a and b). A weak signal for the SMRT 40b + splice variant was observed at the 84 h. hatchling stage probably originating from the developing neural tissue at this time (Fig. 3c).

**Fig. 4:**
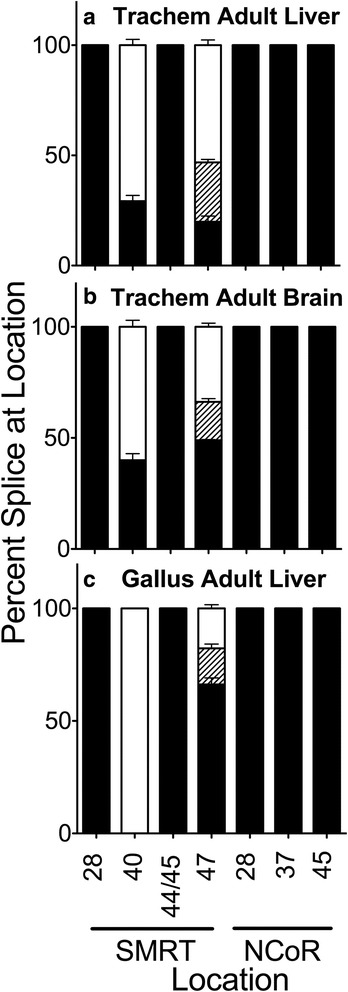
Alternative splicing in *Trachemys scripta* liver and brain, and in *Gallus gallus* adult liver. Adult *Trachemys* liver (**a**), *Trachemys* brain (**b**), or *Gallus* liver (**c**) were isolated as indicated, RNA was extracted, and the alternative splicing at the indicated locations was analyzed and is presented graphically as in Fig. 2; the SMRT exon 47b- variant, of intermediate length between the 47b + and 47- variants, is indicated by diagonal striping. Mean and standard deviation are shown (*n* ≥ 3) for each

SMRT alternative splicing at these sites became more extensive in a reptile, an avia, and an amphibian, whereas NCoR splicing at these sites was not found in these taxa. In common with late stage *Danio* and adult *Danio* brain the SMRT 40b + variant was observed in *Trachemys* (turtle) brain, as well as in *Trachemys* liver, heart, lungs, and kidney (Fig. 4a, b, Additional file 1: Figure S1C, and not shown). We also now detected in *Trachemys* three alternative SMRT splice variants at exon 47 (denoted 47b+, 47b-, and 47-; Fig. 4a and b) but not seen in *Danio*. The exon 47b+/b- alternative splicing is just C-terminal to the RID1 binding motif (Fig. 1) and can dramatically alter the affinity of SMRT for (and/or its ability to mediate repression by) TRs, PPARα, PPARγ, and the liver X receptor (LXR)-α, but not for retinoic acid receptors [22, 23]. The SMRT exon 47- splice variant further removes all of SMRT CoRNR box 1, (Fig. 1) which leads to retention of the affinity of this corepressor for the RARs but which greatly reduces its affinity for most other nuclear receptors tested [22, 23]. None of the other known splice variants of SMRT or NCoR were detected in *Trachemys* by these methods.

A comparable analysis of a bird, (*Gallus gallus*), displayed a mix of the features found in *Danio* and *Trachemys* (Fig. 4c and Additional file 1: Figure S1D). Notably we see very little or none of the SMRT exon 40b + splice variant in the liver, but we do detect significant expression of the SMRT exon 47b+/b−/− alternatively spliced variants. Again no confirmed alternative splicing was observed at any of the other SMRT or any NCoR positions tested.

**Fig. 5:**
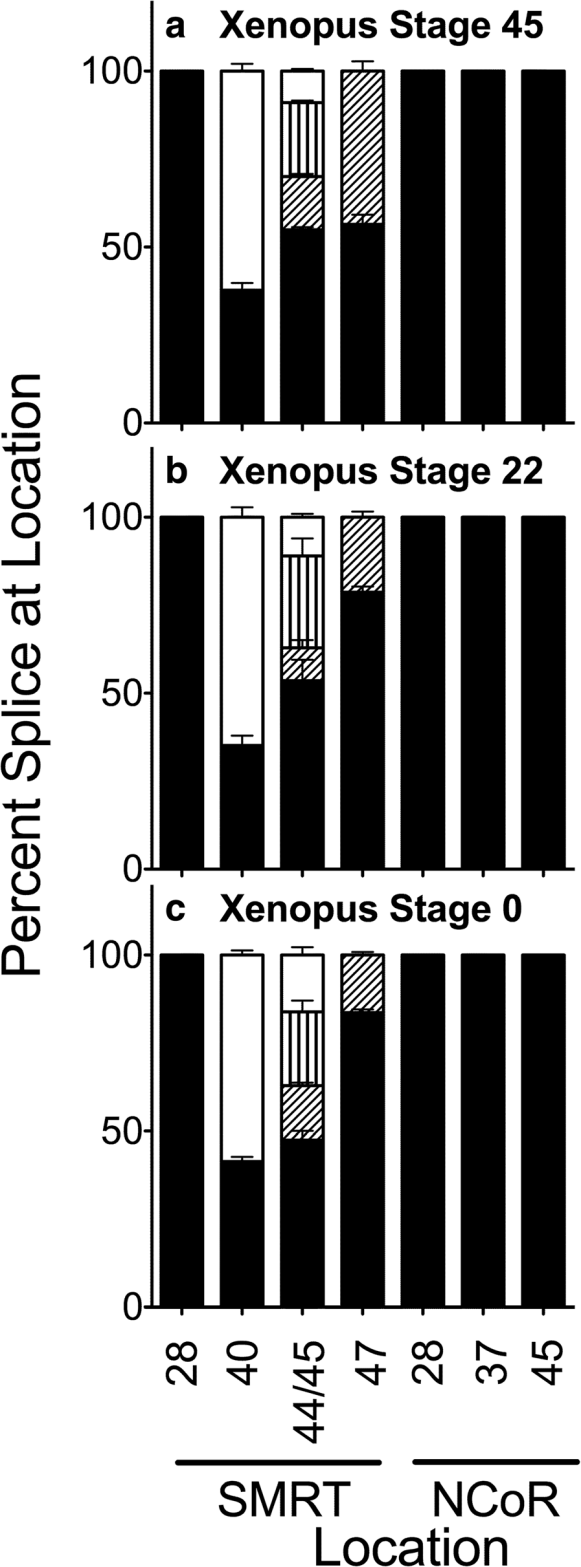
Alternative splicing in whole *Xenopus* at stages indicated. RNA was isolated from entire Xenopus individuals at the stages denoted and the alternative splicing at the indicated locations was analyzed and is presented graphically as in Fig. 2. Fill patterns are as in Figs. 2 and 4 with one exception: four variants are generated from the S44 and S45 positions in this species (S44+/45+. S44+/45-, S44-/45+, and S44-/S45- from largest to smallest; represented by black, diagonal stripe, vertical stripe, and open fill respectively). Mean and standard deviation are shown (*n* = 3) for each

An equivalent analysis of whole *Xenopus* tadpoles (stage 45) revealed a significant amount of the SMRT 40b + splice variant observed in *Danio* as well as the alternative splicing at SMRT exon 47- seen in *Trachemys* and *Gallus* (Fig. 5a and Additional file 1: Figure S1E). However there were several additional features unique to *Xenopus*. For example, *Xenopus* also expressed SMRT splice variants that lacked exon 44, 45 or both (denoted 44-, 45-, and 44-/45-) and that were not observed in any other species studied (see above and below); these alternative splicing events change the spacing between SMRT RID 1 and RID2 although not impinging on the CoRNR motifs themselves in these RIDs (Fig. 1). These changes in spacing are predicted to alter the interaction and function of the encoded corepressor variants when recruited to different transcription factor partners and to different target genes. In addition, although *Xenopus* expressed the SMRT exon 47b- and 47- variants seen in *Trachemys* and *Gallus*, no 47b + splice variant was detected in the amphibian (unlike in the previous two species discussed) (Fig. 5a). Analysis of *Xenopus* also revealed a very small quantity of a PCR product that suggested alternatively splicing at SMRT exon 28 and NCoR 45a+, but more detailed characterization revealed these PCR products were the incorrect molecular weights and were likely to represent PCR artifacts rather than corresponding to one of the authentic alternatively spliced corepressor mRNA previously reported for mice (data not shown). This interpretation is consistent with the reported absence of any alternative splicing at these locations in a prior study of *Xenopus* [25].

Essentially comparable alternative splicing of SMRT and NCoR was observed at two earlier *Xenopus* developmental stages, although some variants were expressed at slightly different relative abundances at the different stages (Fig. 5b and c).

**Fig. 6:**
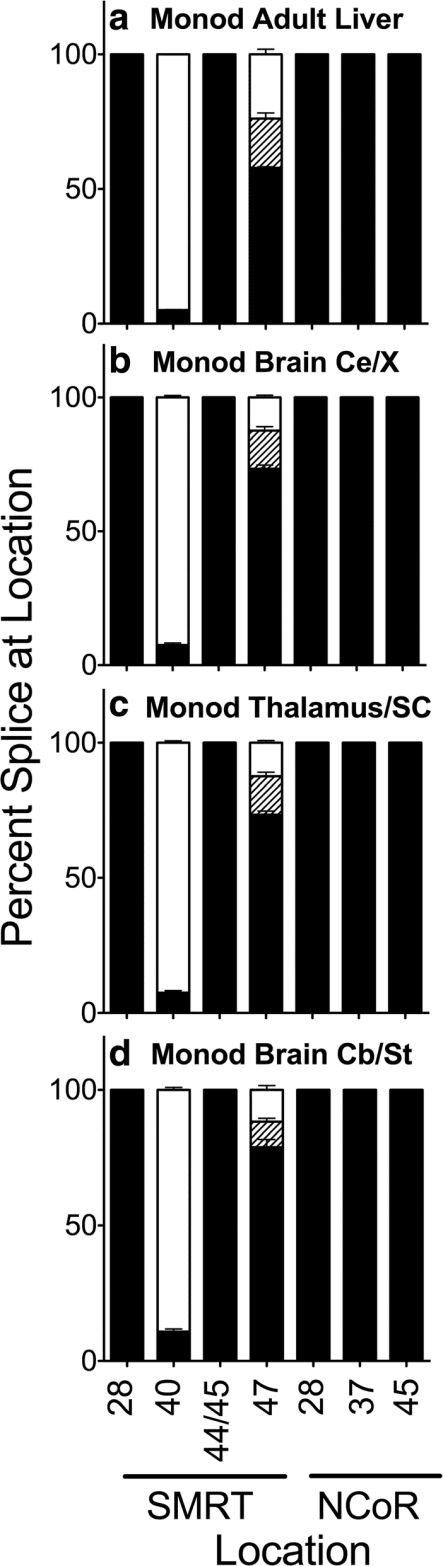
Alternative splicing in adult *Monodelphis domestica* (opossum). Liver (**a**) or the parts of the brain indicated (cerebral cortex (**b**), thalamus/superior colliculus (**c**), and cerebellum/brain stem(**d**)) were isolated by dissection, RNA was extracted, and alternative splicing at the indicated locations was analyzed and is presented graphically as in Fig. 2. Mean and standard deviation are shown (*n* = 3) for each

Corepressor splicing in opossum (*Monodelphis domestica*), a marsupial mammal, also exhibits a mixture of traits compared to that seen in *Xenopus, Trachemys,* and/or *Gallus*. Modest alternative splicing of the SMRT corepressor was also seen in opossum liver (Fig. 6a and Additional file 1: Figure S2A). Alternative splicing to produce SMRT exons 40b+/b- and SMRT 47b+/47b-/47-, as seen in *Trachemys* and late stage X*enopus,* was retained in *Monodelphis*, but no alternative splicing was detected at any other SMRT or NCoR position examined. As already noted, mouse and *Danio* brain expressed higher levels of the SMRT exon 40b + variant than did liver. We therefore also examined alternative corepressor splicing in the *Monodelphis* brain, analyzing cortex, thalamus, and cerebellum/stem regions separately (Fig. 6b-d). The SMRT exon 40b + variant was indeed found at slightly higher abundance in certain regions in *Monodelphis* brain than in liver (although not to the same high enrichment as observed in mouse brain).

**Fig. 7:**
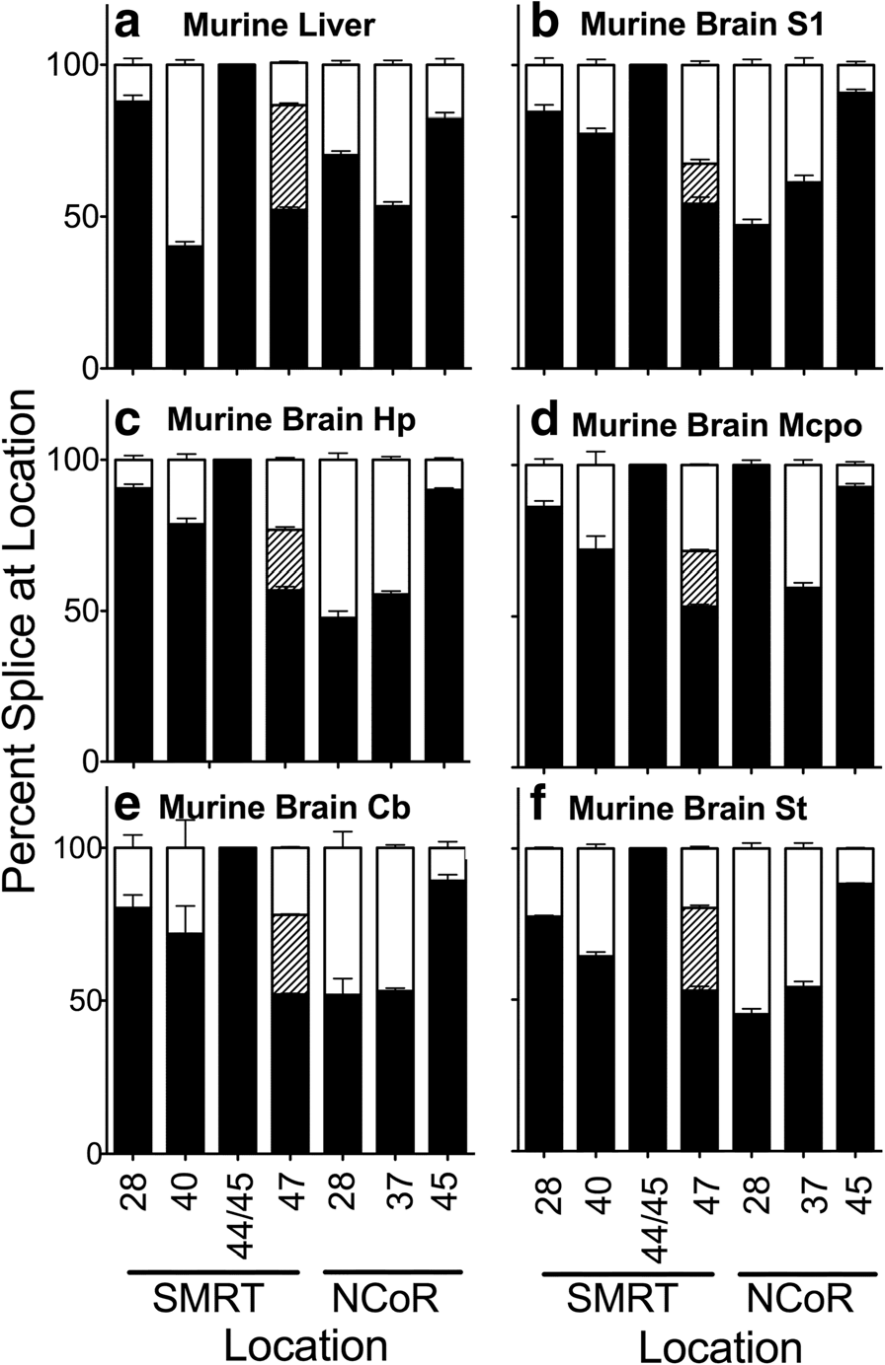
Alternative splicing in adult *Mus mus domestica* (mouse). Liver (**a**) or the parts of the brain indicated: S1 cerebral cortex (**b**), hippocampus (**c**), magnocellular preoptic (**d**), cerebellum (**e**), and brain stem (**f**), were isolated by dissection. RNA was extracted and the alternative splicing at the indicated locations was analyzed and is presented graphically as in Figs. 2 and 4. Mean and standard deviation are shown (*n* = 3) for each

Extensive alternative splicing of both SMRT and NCoR was particularly prevalent among the placental mammals. In common with the above species, we detected in murine liver alternative splicing of SMRT exon 40b+/40b- and SMRT exon 47b+/47b-/47- (Fig. 7 and Additional file 1: Figure S2B). However, we also detected in mouse liver additional alternative splicing events not observed in these other species, yielding SMRT exon 28b+/b-, NCoR exon 28+/−, NCoR exon 37b+/b-, and NCoR exon 45a+/− variants (Fig. 7 and Additional file 1: Figure S2B). In the aggregate these all have the potential to alter assembly of the corepressor complex or interactions of SMRT and NCoR with their transcription factor partners [23]. The SMRT exon 28b+/b- and NCoR exon 28+/− alternative splicing is within or proximal to interaction domains for Sin3, HDAC 4/5, TFIIB, and for the binding sites for the non-receptor transcription factors ETO, Pit-1, PLZF, and Bcl6 in these corepressors [21]. The NCoR exon 37b+/b- alternative splicing event, in common with the previously noted SMRT exon 40b+/b- splicing event, introduces or removes a third CoRNR box/RID domain from the respective corepressor [21]. In mice, these NCoR 37b+/b- variants have opposing effects on lipid metabolism and alternative spicing at this site is in turn regulated by both diet and by pro-adipogenic drugs [24, 29]. The NCoR exon 45a+/− alternative splicing event inserts or removes a C-terminal exon that overlaps or is proximal to interaction sites for Pit-1, PLZF, Bcl-6, NF-kb, SRF, Oct-1, Sharp, and TF-IIB [21]. Interestingly the alternative splicing of exon SMRT 44 and 45 seen in *Xenopus* that alters the spacing between RID 1 and 2 appears to be unique to *Xenopus* (compare Figs. 2, 3, 4, 5, 6 and 7).

We next examined these alternative splicing events in different regions of mouse brain: S1 cortex, hippocampus, and magnocellular preoptic nucleus (a subregion of the mouse thalamus) (Fig. 7). The locations of the alternative splicing sites in mouse brain paralleled those observed in mouse liver (Fig. 7), although we did see the enrichment of the SMRT exon 40b + and NCoR exon 37b- splice variants in these subregions as previously noted for whole brains. Very similar results were also obtained with mouse brain dorsal thalamus and hypothalamus (Additional file 1: Figure S2C and data not shown).

**Fig. 8:**
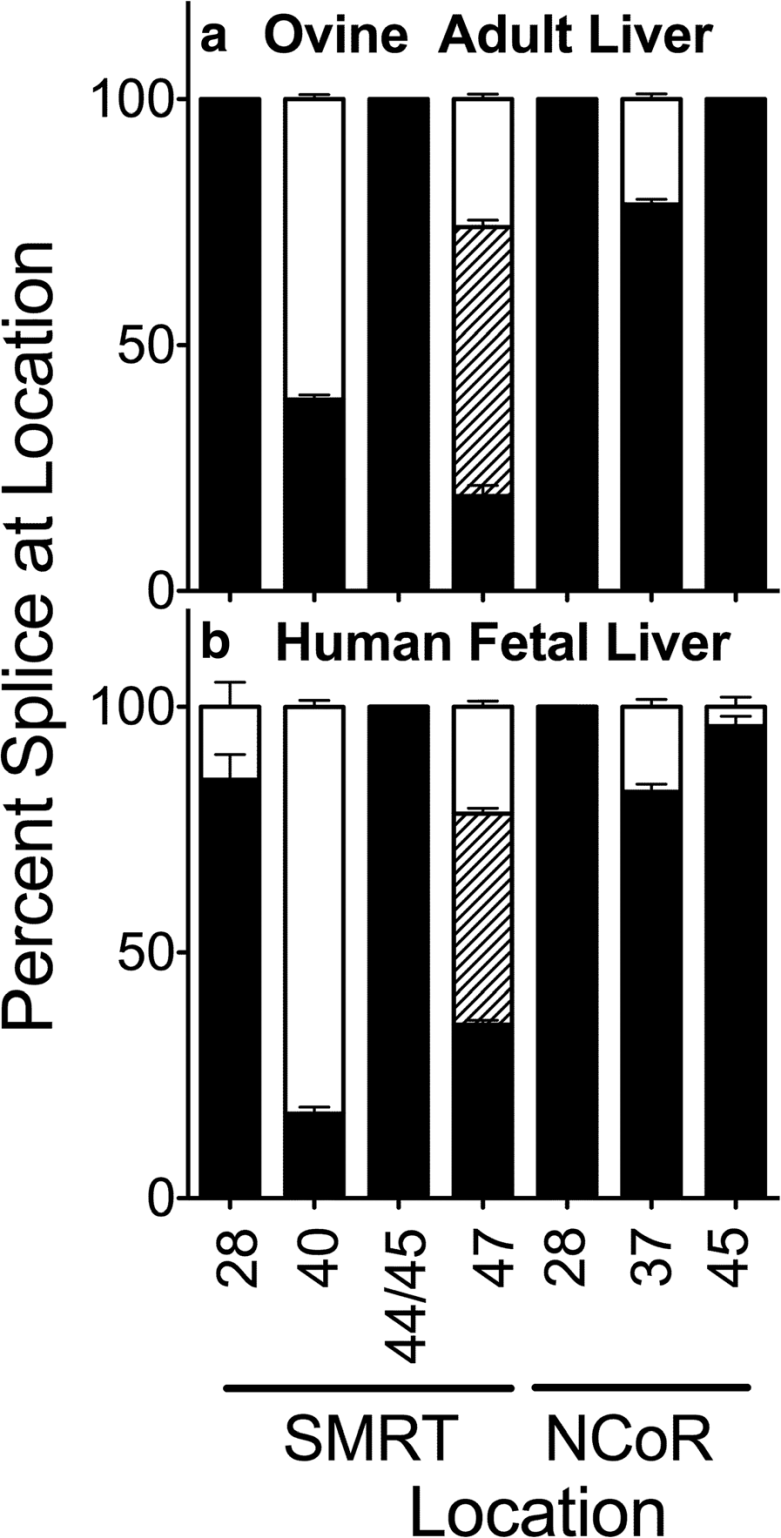
Alternative splicing in ovine and human liver. Livers were isolated, RNA was extracted, and the alternative splicing at the indicated locations was analyzed and is presented graphically as in Figs. 2 and 4. Mean and standard deviation are shown (*n* = 3) for each. Panels represent ovine adult liver (**a**) and human fetal liver (**b**)

We expanded these studies to characterize alternative corepressor splicing in sheep and in humans. In both the *Ovine* and *Homo* samples we detected alternatively spliced variants of SMRT at exons 40b+/40b-, 47b+/47b-/47- and NCoR at exon 37b+/37b-, as seen in mouse (Fig. 8). There was no evidence for the SMRT exon 44- and 45- alternative splicing, again indicating that these modifications were limited to *Xenopus*. However, there were some differences among the placental mammals: the SMRT exon 28b- and NCoR exon 45a- variants seen in mice were found in the human but not the ovine samples, whereas the NCoR exon 28- splice variant was not detected in either the human or ovine liver materials.

Molecular basis of taxonomic variation in alternative corepressor splicing. What was the molecular basis behind the differences of alternative corepressor splicing observed among the species examined? In several species the absence of detectable splice forms was consistent with the absence of appropriate *cis*-acting consensus splice donor sequences from the corresponding splice sites (such as the absence of an appropriately located GT donor sequence that would be required to generate a *Danio* NCoR exon 37b + splice variant [39]). In many other species, however, the necessary splice donor or acceptor sequences are present [39] yet alternative splicing originating from these sites was not observed in practice. Presumably a lack of appropriate *trans*-acting factors in these species, or alterations of yet-unidentified *cis*-sequences within the relevant introns, account for this absence of alternative splicing at these locations. Differences in *trans*-acting factor expression might also account for the differences in corepressor alternative splicing within different tissues and at different developmental stages in the same species.

Alternative splicing of these corepressors at other mRNA sites and in non-vertebrates. Our above studies were focused on the alternative splicing events previously reported to occur in *Xenopus* and/or mice (the splicing events that altered the function or presence of the RID domains were of particular interest). To explore if there might be alternative splicing at yet additional locations in certain species but not previously observed in the *Xenopus* and mammalian studies, we next employed additional PCR primers that surveyed the entire SMRT and NCoR open reading frames, and thus would detect any previously undescribed alternative splicing. We initially studied *Danio* given that this telostat is the most taxonomically distant from the species employed in our prior published studies.

Notably in *Danio* there was evidence of additional alternative splicing events located between NCoR bases 2589 and 3594 and SMRT bases 3134 and 3978 (Additional file 1: Figure S3, NCoR primer pair 3 and SMRT primer pair 4). The ratios of the alternatively spliced variants at these locations differed between different *Danio* organs (e.g., Additional file 1: Figure S3A and B). These *Danio* alternative splice sites were not reported in prior studies of mammalian or *Xenopus* alternative splicing. They map to an NCoR region that that bears a domain implicated in the Siah2-mediated degradation of NCoR, the binding of unacetylated histone H4, and to a SMRT region previously shown to contribute in an unresolved manner to the overall repression phenotype [21, 40]. In addition to these novel SMRT and NCoR variants, the alternative splicing of SMRT exon 40b+/b- already described above for *Danio* brain was again evident when using the extended SMRT survey primer pairs 7 or 8 (which overlap the relevant region; Additional file 1: Figure S3A). The biological consequences of these newly recognized alternative splicing events will be examined in future experiments.

To further extend this type of analysis beyond vertebrates, we next examined the alternative splicing of SMRTER in the arthropod *Drosophila melanogaster*. SMRTER, and its nematode ortholog GEI-8, bear notable, although limited, sequence relatedness to the deuterostoma SMRT and NCoR corepressors (e.g., [19]). By surveying the *Drosophila* SMRTER open reading frame as above, we identified two regions, between nucleotide 6275–6779 and between 6760 and 7302, that were alternatively spliced (Additional file 1: Figure S4). The former removed approximately 500 bps and the second added approximately 150 bps. There is no observable sequence relatedness between these alternatively spliced regions in SMRTER and the vertebrate SMRT or NCoR, leaving the functional impact of these alternative splices difficult to predict. Nonetheless we conclude that alternative splicing of the SMRT/NCoR/SMRTER family exists widely throughout phylogeny, but differs in its details across different taxa.

## Discussion

NCoR and SMRT are thought to have arisen from an ancestral duplication and divergence of a common precursor locus, with this divergence occurring just prior to the evolutionary appearance of vertebrates. Distinct loci for NCoR and for SMRT (also known as NCoR1 and NCoR2) can be identified in virtually every vertebrate genome sequenced to date, spanning phylogeny from the cartilaginous and bony fish to the primates (e.g., [17, 39]). There is an approximate 36 % overall amino acid identity between NCoR and SMRT, and NCoR and SMRT play both unique and shared roles in biology. Loci with extended sequence relatedness to both NCoR and SMRT also exist in the genomes of the non-vertebrate Chordata and Echinodermata, but apparently as a single “combined” locus in each genome [17]. Therefore the initial duplication and divergence of distinct SMRT and NCoR corepressor loci likely occurred early during the origins of the vertebrates [17, 21]. Whether this was a localized gene duplication or reflected instead the more extensive whole genomic duplications proposed for early vertebrate evolution in the “2R” hypothesis [41] is unclear, although a preliminary chromosomal synteny search appears to favor the former hypothesis (data not shown).

Even more distant phyla, such as the arthopoda and nematoda, also contain single loci (e.g., SMRTER and GEI-8) related to both vertebrate SMRT and NCoR. However the sequence relatedness of these more distant loci is limited to N-terminal regions, and lacks the C-terminal architecture and CoRNR box sequences characteristic of deuterosomatia NCoR and SMRT [19]; thus the precise evolutionary or functional relationship of the former to the latter is not clear.

Alternative splicing of SMRT occurs in the teleost *Danio*, in the bird *Gallus*, and in the reptile *Trachemys*, but this is relatively minimal at the RNA sites previously reported in mammals or amphibians. A very modest amount of production of the SMRT exon 40b + variant was detected in *Danio* at the 84 h stage (i.e., “hatchlings” that display an overall adult-like morphology). Dissection of *Danio* adults indicates that this 40b + SMRT variant is found primarily in the brain, in parallel to the enhanced abundance of this variant in the murine brain. The *Danio* SMRT 40b + variant however represents only a small fraction of the total SMRT transcripts in brain and was not detected in any other organ examined. The 40b + SMRT exon contains an S3-CoRNR box whereas the 40b- exon does not. Thus the 40b + exon SMRT variants can encode up to three RIDs, whereas the 40b- exon SMRT variants contains no more than two RIDS. The murine SMRT40b+/b- variants are known to display greatly different preferences for different nuclear receptors and to play distinct biological roles [20, 22, 23, 26, 27]. In contrast, no alternative spliced variants at SMRT at exons 28b + or 47b-, or at NCoR exons 28+, 37b, or 45a, are detected in *Danio*.

Corepressors in the bird *Gallus* also display limited alternative slicing, but at SMRT exon 47 (producing 47b+, b-, and – variants) and not at SMRT exon 40b. We have shown that proteins derived from the three different SMRT exon 47 variants also possess distinct affinities for different nuclear receptors [22]. Alternative splicing in the reptile, *Trachemys*, exhibits both the SMRT exon 40b+/b- and SMRT exon 47b+/b−/− splice variants, which further diversify the potential functions of SMRT in these taxa compared to *Danio or Gallus*.

Both shared and unique patterns of alternative splicing of SMRT are observed in the amphibian, *Xenopus*. A high abundance of the 40b + SMRT variant is observed in *Xenopus*, as well as the additional alternative splicing event at SMRT exon 47. Interestingly, although all three exon 47 variants are expressed in *Gallus* and *Trachemys* the exon 47- variant is lacking in *Xenopus*. Similarly, alternatively spliced SMRT variants which alter the distance between RID1 and 2 (exon 44+/− and 45+/−) are observed in *Xenopus* but are not seen in any other lineage we examined (these too are anticipated to impact on SMRT affinity for its different transcription factor partners). Analogous patterns of alternative splicing sites is also observed at earlier stages of *Xenopus* development.

We suggest that the divergence of amphibia, reptilia, and ava from the teleost lineage was coincident with a more extensive use of the SMRT 40b + exon and/or additional alternative splicing at SMRT exon 47, both of which significantly diversify the biochemistry and transcription partner preferences displayed by this corepressor. Notably these three lineages express nuclear receptors, e.g., TRs, PPARs, and LXRs, that have been shown to exhibit different interactions with the corresponding corepressor splice variants (as tested in vitro using the mammalian versions) [22, 23].

The alternative splicing of NCoR is restricted to placental mammals and appears to have arisen later in evolution than that of SMRT. Alternative splicing of SMRT was observed in both the placental and marsupial lineages examined, although to different extents among the different species. However unlike SMRT, alternative splicing of NCoR was not observed in non-placental mammals; of the 3 different NCoR alternative splice sites that are utilized in the placental mammals, the alternative splicing of NCoR at exon 37b-/b + is of particular interest. Although restricted to the placental mammals, this alternative splicing of NCoR alters the structure of the encoded corepressor in a manner remarkably similar to the alternative splicing that occurs in virtually all vertebrates at SMRT exon 40b+/b-. Both the NCoR and SMRT splicing events insert or remove a third RID region from the encoded protein, both display closely related tissue distributions (including particularly high abundances of the RID3-containing variants in brain and testis), and both produce proteins with overlapping preferences for certain transcription factor partners [23, 26, 27].

Despite these commonalities, however, the precise sites of the splices, the length of the alternative spliced exons, and the precise amino acid compositions of these exons differ substantially between NCoR and SMRT [39]. Although we do not rule out the possibility that this alternative splicing occurred prior to the SMRT/NCoR divergence and that NCoR subsequently lost this ability in several lineages [17], we view the data overall as favoring instead the proposal that the NCoR N37b+/b- and SMRT S40b+/b- alternative splice sites arose separately at different times in evolution and are the outcome of a convergent evolutionary process. We speculate that this convergent evolution may have been driven by functional requirements unique to the placental mammals that favored diversification of NCoR into RID3 −/+ variants in a manner similar, but operating in addition, to that which favored diversification of SMRT into its RID3−/+ variants during earlier vertebrate evolution [23]. Consistent with this hypothesis, NCoR 37b+/b- splicing regulates adipose tissue function, lipid accumulation, and glucose utilization in ways that SMRT 40b+/b- splicing does not [24, 29]. The effects of diet on NCoR 37b+/b- and SMRT 40b+/b- splicing also differ [24, 29]. Given known differences in these energy storage and utilization processes in placental mammals versus other vertebrates, it is tempting to speculate that the acquisition of alternative splicing at the NCoR 37b+/b- site in placental mammals may in part reflect a requirement for more sophisticated regulation of these metabolic pathways in the former.

Also of relevance: SMRT is alternatively spliced at exons 47b+/47b-/47- so as to alter the presence and function of its RID 1 domain [22], yet no comparable alternative splicing is detected in NCoR in any lineage examined. The acquisition of this alternative splicing event by SMRT but not by NCoR is therefore likely an example of a divergent, rather than convergent, evolutionary event that occurred after duplication of the common ancestral corepressor gene.

Novel sites of alternative splicing of SMRT and NCoR in different species and in *Drosophila* SMRTER. We also detected previous undescribed alternative splice sites within the coding regions of both *Danio* SMRT and NCoR, as well as of *Drosophila* SMRTER. These additional alternative splicing sites map outside of the RIDs themselves and affect regions that may influence the assembly of the corepressor holocomplex, histone binding, or functions yet to be established. Our results therefore indicate that alternative corepressor splicing is a common theme among a wide variety of metazoans with utilization of these splice sites differing from species to species.

Limitations of our methodology. As detailed in the Materials and Methods, our approach was highly reproducible, with little or no variation detected among different individuals within a species. Our methodology did require sufficiently annotated genomic sequences to allow design of RT-PCR primers, as well as a source of adequately intact RNA. As a result, several taxa that might have extended our analysis, such as monotremes, were unavailable to us, and we were restricted to examining only a limited number of species from a given taxonomic order. Certain corepressor splice variants may only exist within specific tissues or organs that were not tested in this study (although we typically see greater differences in the sites of alternative splicing between species than between the different tissues/organs within a single species; this manuscript and data not shown). Finally we can not fully rule out the presence of splice variants that might be produced below detectable levels (~2 % or less) or that might lie outside the specific regions analyzed here.

Despite these limitations, our experiments nonetheless elucidated key aspects of the evolution of the previously-reported alternative splice sites in these corepressors, particularly the alternate splicing events within the RIDs that define the interactions of these corepressors with many of their transcription factor partners. Full understanding of the biological implications of the novel corepressor splice sites newly detected by our experiments in *Danio* and in *Drosophila* will require extensive future biochemical and taxonomic analysis.

## Conclusions

**Fig. 9:**
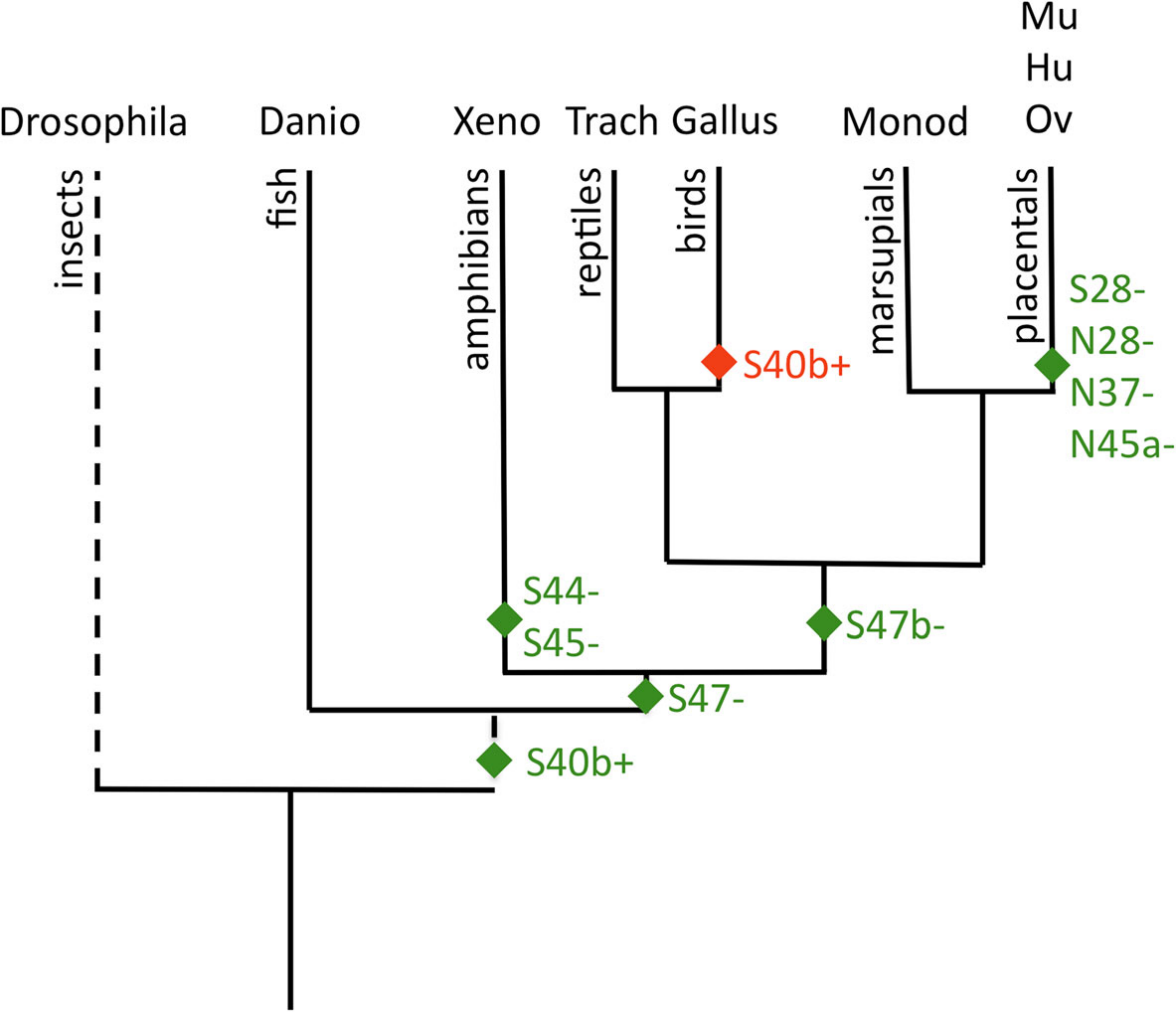
Evolution of alternative splicing for SMRT and NCoR. A dendrogram representation of evolutionary divergence is presented for the species analyzed at the alternatve splice sites previously reported. Dashed lines indicates use of different time scale for the *Drosophila* divergence. The postulated gain (*green symbols*) or loss (*red symbol*) of each alternative splicing site is depicted near the base of the relevant lineages (S = SMRT and *N* = NCoR]. A tissue-specific dendrogram would differ only by the absence of detectable S40 alternative splicing in *Danio* tissues other than brain and whole 84 h. hatchlings; otherwise the different tissues within a given species vary in the abundance of the alternative spliced variants noted here, but not in their presence or absence

Corepressor alternative splicing appears to be an ancient strategy and is detected in lineages that diverged some 500 Ma ago, but that became more elaborate during the vertebrate radiation ~100 Ma ago. Alternative corepressor splicing most likely first appeared at the exon 40 position in SMRT very early in vertebrate evolution, with alternative splicing at the SMRT exon 47 and 28 positions appearing during subsequent tetrapod and placental mammal evolution, respectively (Fig. 9 and Additional file 1: Figure S5). In contrast, alternative splicing of NCoR at any of the positions examined (exons 28, 37, or 45a) probably first appeared significantly later and only during early placental mammal evolution (Fig. 9 and Additional file 1: Figure S5).

It is notable that alternative splicing, which is frequently viewed as a “polishing” step in gene expression that only modestly alters function, has in fact dramatic, even opposing effects on corepressor function. Splicing therefore appears to be a fundamental process by which a limited number of genes are diversified to yield distinct protein products that exert distinct functions in different tissues and at different times in development and differentiation. This manuscript reveals that different species employ different strategies in their alternative splicing of SMRT and of NCoR. Rather than viewing this as a neutral variation we favor the interpretation that this species-specific utilization of alternative corepressor splicing reflects natural selection and serves to customize alternative corepressor splicing to the biological requirements of each species. We base this view on several observations. (a) The alternative splice sites are located non-randomly such that they incorporate or exclude many of the important functional domains previously identified within the corepressor open reading frame. As a consequence the individual corepressor splice variants exhibit different molecular properties, favor different transcription factor partners, and play different roles in essential biological processes. The ability of a species to express a given alternative corepressor splice variant is therefore expected to represent an important selectable trait. (b) Consistent with this concept, the relative abundances of the different splice variants within a given species are actively controlled in response to tissue type, differentiation signals, and extracellular environment, further suggesting that the different variants exert biologically significant, yet distinct, functions that are coordinated so as to maintain the overall health and fitness of the organism. (c) Alternative splicing that adds or removes the S3/N3 RIDs appears to have arisen separately in SMRT and NCoR by convergent evolution, suggesting that these alternative splicing events were under selective pressure so as to generate similar (but not identical) variants of each corepressor paralog.

## Additional file

Additional file 1:Figure S1. Electrophoretograms depicting alternative splicing in *Danio*, *Trachemys*, *Gallus*, and *Xenopus*. Alternative mRNA splicing was analyzed for each species by PCR as described in the Materials and Methods; photographs of representative ethidium bromide-stained electrophoretograms (as employed for our bar-graph quantifications) are shown. (A). *Danio* liver. (B) *Danio* whole 84 h. stage hatchling. (C) *Trachemys* liver. (D) *Gallus* liver. (E) *Xenopus* liver. The splice site analyzed is indicated below each lane; colored bars next to the stained DNA bands in each lane identify the splice variants in that lane from largest to smallest at that location (red bars therefore indicate S28+, S40b+, S44+/45+, S47b+, N28+, N37b+, or N45a+; blue bars indicate S28-, S40b-, S44+/45-, S47b-, N28-, N37b-, or 45a-; green bars indicate S44-/45+ or S47-. black bars indicate S44-/45-). “Background” faint DNA bands (not labeled with bars) likely resulted from artifactual cross-hybridization of given PCR primer pairs to irrelevant nucleic acid sites (confirmed by their absence in follow-up PCR analysis using novel primer pairs and/or by DNA sequence analysis). **Figure S2.** Electrophoretograms depicting alternative splicing in *Monodelphis* and *Mus*. The analysis and labeling are as in Additional file 1: Figure S1. (A). *Monodelphis* liver. (B) *Mus* liver. (C) *Mus brain hypothalamus*. **Figure S3.** Extended survey of alternate splicing in *Danio rerio*. Primer pairs (detailed in Additional file 1: Table S1) were used to survey potential alternative splicing within the open reading frame of SMRT and NCoR. Brain (A) and liver (B) RNAs were tested. Primers 7 and 8 amplify a partially overlapping region that includes the SMRT 40b+/b‐ alternative splicing already noted in Figs. 2 and 3. Black fill = longest splice variant at that location, diagonal hatching = intermediate length splice variant at that location, open fill = shortest splice variant at that location. Mean and range are shown (n = 2) for each. **Figure S4.** Alternative splicing in *Drosophila melanogaster* adults. RNA was extracted, and the alternative splicing at the indicated locations was analyzed using an ordered series of primer pairs that encompass the known coding region (Additional file 1: **Table S1** and Fig. S2). No more than two spliced products were detected at any given location. Mean and standard deviation are shown (n = 3) for each. **Figure S5.** Gene-tree depiction of alternative splicing for SMRT and NCoR. Vertical distance is proportional to the minimal number of events to generate the alternative splicing detected in each lineage at the sites depicted. Each alternative splicing event is labeled as S (SMRT) or N (NCoR) with the number and −/+ symbols indicating the specific exon variant (as in Fig. 1); green text indicates the appearance of that specific splice variant, red text indicates its loss. Vertical boxed black text above each terminal arrow indicates the alternative splice sites utilized in each of the extant species studied here, with the species listed on top in blue; the corepressor configurations predicted in the absence of alternative splicing are indicated in horizontal boxed black text: (N28+,N37b+,N45a + and S28+,S40-,S44+,S45+,S47b+). The presence of an alternative splice variant in a given species was scored as a positive if detected in any of the tissues or whole organism samples used in this study. In all but *Danio* a given alternative splice variant was present or absent in all the developmental and tissue samples tested for that species (although at differing relative abundances). (PDF 14424 kb)
