# Supplementary material for: Corepressor diversification by alternative mRNA splicing is species specific
Source: BMC Evol Biol. 2016 Oct 19;16:221. doi: 10.1186/s12862-016-0781-2 (PMC5069798; doi:10.1186/s12862-016-0781-2)
Supplement: Additional file 1:Figure S1. — Electrophoretograms depicting alternative splicing in Danio, Trachemys, Gallus, and Xenopus. Alternative mRNA splicing was analyzed for each species by PCR as described in the Materials and Methods; photographs of representative ethidium bromide-stained electrophoretograms (as employed for our bar-graph quantifications) are shown. (A). Danio liver. (B) Danio whole 84 h. stage hatchling. (C) Trachemys liver. (D) Gallus liver. (E) Xenopus liver. The splice site analyzed is indicated below each lane; colored bars next to the stained DNA bands in each lane identify the splice variants in that lane from largest to smallest at that location (red bars therefore indicate S28+, S40b+, S44+/45+, S47b+, N28+, N37b+, or N45a+; blue bars indicate S28-, S40b-, S44+/45-, S47b-, N28-, N37b-, or 45a-; green bars indicate S44-/45+ or S47-. black bars indicate S44-/45-). “Background” faint DNA bands (not labeled with bars) likely resulted from artifactual cross-hybridization of given PCR primer pairs to irrelevant nucleic acid sites (confirmed by their absence in follow-up PCR analysis using novel primer pairs and/or by DNA sequence analysis). Figure S2. Electrophoretograms depicting alternative splicing in Monodelphis and Mus. The analysis and labeling are as in Additional file 1: Figure S1. (A). Monodelphis liver. (B) Mus liver. (C) Mus brain hypothalamus. Figure S3. Extended survey of alternate splicing in Danio rerio. Primer pairs (detailed in Additional file 1: Table S1) were used to survey potential alternative splicing within the open reading frame of SMRT and NCoR. Brain (A) and liver (B) RNAs were tested. Primers 7 and 8 amplify a partially overlapping region that includes the SMRT 40b+/b‐ alternative splicing already noted in Figs. 2 and 3. Black fill = longest splice variant at that location, diagonal hatching = intermediate length splice variant at that location, open fill = shortest splice variant at that location. Mean and range are shown (n = 2) for each. Figure S4. Alt [file 12862_2016_781_MOESM1_ESM.pdf]

Supplemental Table S1: Oligonucleotides Used As Primers for RT-PCR			
**Organism**	**Location**	**Up Primer**	**Down Primer**
Chrysemys	NCoR28+/-	cagccaaaggccatgttatt	ggtcctgtcttgggatttca
	NCoR 37b+/b-	ggatcctgctgctcagctac	ttcttggggaggtacaggtg
	NCoR 45a+/a-	agcctatggggatagcacct	caatggacgttggtggtgta
	SMRT 28+/-	ctccccctctcatctcttcc	tttggtcaaggcatcctctc
	SMRT 40b+/b-	tagcagaggacaggggaaaa	tactgcttcaacgccatcag
	SMRT 44+/-/45+/-	agaacatcaaaggccaccag	ttgctggcatgttgaaaatc
	SMRT 47b+/b-/-	gattttcaacatgccagcaa	cccagactcgactggttagc
Danio	NCoR28+/-	gccacagaggaccagaacag	agagcgtatgggtggactct
	NCoR 37b+/b-	ccgatacaacaacgctgctg	ggagagtttgctgggctgat
	NCoR 45a+/a-	ggggaacatggaggagaagc	gcatcgtcaggggggttgtaa
	SMRT 28+/-	ggctccatcacacagggtac	gagacgggttattgtgccct
	SMRT 40b+/b-	agcgcgtcaagtagggaaaa	ctggttttgctgtgactggc
	SMRT 44+/-/45+/-	cagagggcacttctgcagaa	ttctgtcccaggttggctct
	SMRT 1	gaggtcggtgactaccagga	gctcctgcagctctctctgt
	SMRT 2	gaggcctgggagaagaaagt	ttaccactgactcccgatcc
	SMRT 3	tcggagaaagagcgtaaagc	ctctggcaaaatgggaggta
	SMRT 4	tacctcccattttgccagag	gagacgggttattgtgccct
	SMRT 5	ggctccatcacacagggtac	ctcgatctttctcacgctcc
	SMRT 6	ctgtcccgtgtctctccatt	ctcgatctttctcacgctcc
	SMRT 7	ggagcgtgagaaagatcgag	ctggttttgctgtgactggc
	SMRT 8	agcgcgtcaagtagggaaaa	ttctgtcccaggttggctct
	SMRT 9	agagccaacctgggacagaa	ggaggtatcagagggcatga
	NCoR 1	aaggacatgaggtggtttgg	attgttctcctgctccgaga
	NCoR 2	tctcggagcaggagaacaat	cgtcactctcctcatcgtca
	NCoR 3	tgacgatgaggagagtgacg	attactgccatcaggttggg
	NCoR 4	cccaacctgatggcagtaat	agagcgtatgggtggactct
	NCoR 5	gccacagaggaccagaacag	gaatgccctaggagggaaag
	NCoR 6	ctttccctcctagggcattc	ggagagtttgctgggctgat
	NCoR 7	cgcagatggatgtagcaaaa	gtcgctgtctgagagggtct
	SMRT 47b+/b-/-	agagccaacctgggacagaa	ccaggatgtgttggggaagg
Gallus	NCoR28+/-	cgagaggaagcttcagccaa	ggctccttcgaatgccctta
	NCoR 37b+/b-	cttcccgctacaatacggct	tctgcctggcttggtttagg
	NCoR 45a+/a-	gacgacaagagcgaggatca	tactgctgagcatccgcatt
	SMRT 28+/-	gccactgacaatggatccca	tcactttctccgctctgctg
	SMRT 40b+/b-	ggagtcgaggcctttacctg	ccctttctcgtgggataggc
	SMRT 44+/-/45+/-	atcaccctcagcagctcaac	aaatctctgtcccaggctgc
	SMRT 47b+/b-/-	gcagcctgggacagagattt	cccctctgagtgcactgaag
Homo	NCoR28+/-	aggctttggtgaaggggtcca	tcagttgttgctcttggtgtc
	NCoR 37b+/b-	acagagacccagtgttttccaag	gcaggacttatcacctcaatagca
	NCoR 45a+/a-	ccagtaatcttgggctggaa	ccgacagggtctcgtactgt
	SMRT 28+/-	aatgtcggtccagctccacgt	cctttggtgatgcttccgcc
	SMRT 40b+/b-	cggacccgcaccgggaaaagac	aggtgcttggggagccccttg
	SMRT 44+/-/45+/-	aggggtcaaaggtcaccag	gatggcgggcatattgaag
	SMRT 47b+/b-/-	caagaagctgaacacccaca	cgagtgcactgaggagacag
Monodelphis	NCoR28+/-	ccaaaggccacgtgatttat	acgtcccatttctttgatgg
	NCoR 37b+/b-	ccctcagatggaagtgacca	cattccttctgcttgggaag
	NCoR 45a+/a-	ttgtcttggcacaacctctg	tgagtgactgcttggctcac

	SMRT 28+/-	gagttccaatccagctccac	cctggtgatggttcctttgt
	SMRT 40b+/b-	gcaccgagacaaaactccag	gtgatgacctcgctgatgtg
	SMRT 44+/-/45+/-	tgccctgtgctggacttac	ggccggcatgttgaatatct
	SMRT 47b+/b-/-	cagcctggaacggagatatt	ggtcctgcggttacagtctc
Mus	NCoR28+/-	gccatgttatctatgaaggcaaaa	agtcctctctttgaggtcagaatg
	NCoR 37b+/b-	ctggctgctcttgtggatgc	ctgtcccattccctctgactg
	NCoR 45a+/a-	ttcttttgctgatcccgcca	gcatccgtatggtcagaggg
	SMRT 28+/-	gatgtcagtccagcttcgtgtg	cccttggtgatgcttccact
	SMRT 40b+/b-	ccatccaggaattggaactccg	tcaccgggctgatgggctc
	SMRT 44+/-/45+/-	ccacccagtgacctctacct	agtgatggcgggcatgtt
	SMRT 47b+/b-/-	aacaagaaactcaacacccacaac	ctgcctgtagcctcataatcaaag

Ovis	NCoR28+/-	tgaggcattggtgaagggac	ctcttggtgtgccatgcatg
	NCoR 37b+/b-	ctgctccccagatggatgtg	agtgatgcagtggtccttcg
	NCoR 45a+/a-	caaagtggaggatcacgggg	actgtgtcgagcctgttgag
	SMRT 28+/-	ccacgtcccatactcagagc	tcctcaccgatgatcctggt
	SMRT 40b+/b-	tgcaccgggaaaagactca	ctcttgtcgagctcctccag
	SMRT 44+/-/45+/-	ccccctctactccttcccc	tgaagatctctgtccccggt
	SMRT 47b+/b-/-	accggggacagagatcttca	ggttgtaggggaatggggtg
Xenopus	NCoR28+/-	ggagaggtgcagggaggata	gccttttgagatggctccct
	NCoR 37b+/b-	attggccgcacttgttgatg	aggagactcttgctgttgcc
	NCoR 45a+/a-	cccctaacagtgaagcacgt	ggacatagaggatggcggtg
	SMRT 28+/-	cccacttcacgttcctt	tatgacatgccctttggaca
	SMRT 40b+/b-	ttctcccattaaggctgtgg	gtccaatcctgagctttcca
	SMRT 44+/-/45+/-	tatactggagccggttgtcc	cctgtattggctggcatgtt
	SMRT 47b+/b-/-	aacatgccagccaatacagg	tgcactgatgaagcagaggg
Drosophila	SMRTER 1	tctcggtcttcttgctgagg	atggcgggtaaccaggattg
	SMRTER 2	caatcctggttacccgccat	ccagactcccgaccattacg
	SMRTER 3	cgtaatggtcgggagtctgg	gtctccaccttgagctgcat
	SMRTER 4	atgcagctcaaggtggagac	ccgattggccgcatagatct
	SMRTER 5	agatctatgcggccaatcgg	ggccatccatgatctcctcg
	SMRTER 6	cgaggagatcatggatggcc	cttttcggcggcactttcag
	SMRTER 7	ctgaaagtgccgccgaaaag	cagtcgctgtttccggtttg
	SMRTER 8	ctgaaagtgccgccgaaaag	cagctcccaaaccctcaact
	SMRTER 9	agttgagggtttgggagctg	cacaaagcagctggccaatt
	SMRTER 10	aattggccagctgctttgtg	aacggaagcggtatcggatc
	SMRTER 11	gatccgataccgcttccgtt	agctccacctggttgaacac
	SMRTER 12	gtgttcaaccaggtggagct	cggatgaccaattcccacca
	SMRTER 13	tggtgggaattggtcatccg	tacatgccaccagagcgtac
	SMRTER 14	gtacgctctggtggcatgta	cctgaccaagccatcgaact
	SMRTER 15	agttcgatggcttggtcagg	cgtgttgtgcctctggatga
	SMRTER 16	tcatccagaggcacaacacg	ccacctgatccagtgaccac
	SMRTER 17	gtggtcactggatcaggtgg	atatggtggccgcagatgag
	SMRTER 18	ctcatctgcggccaccatat	ctccggatccaggacctc
	SMRTER 19	gaggtcctggatccggag	gtggatgatacggcgaatgg
	SMRTER 20	cacctatgccttcccctaca	ctactgctattgtcgctggc

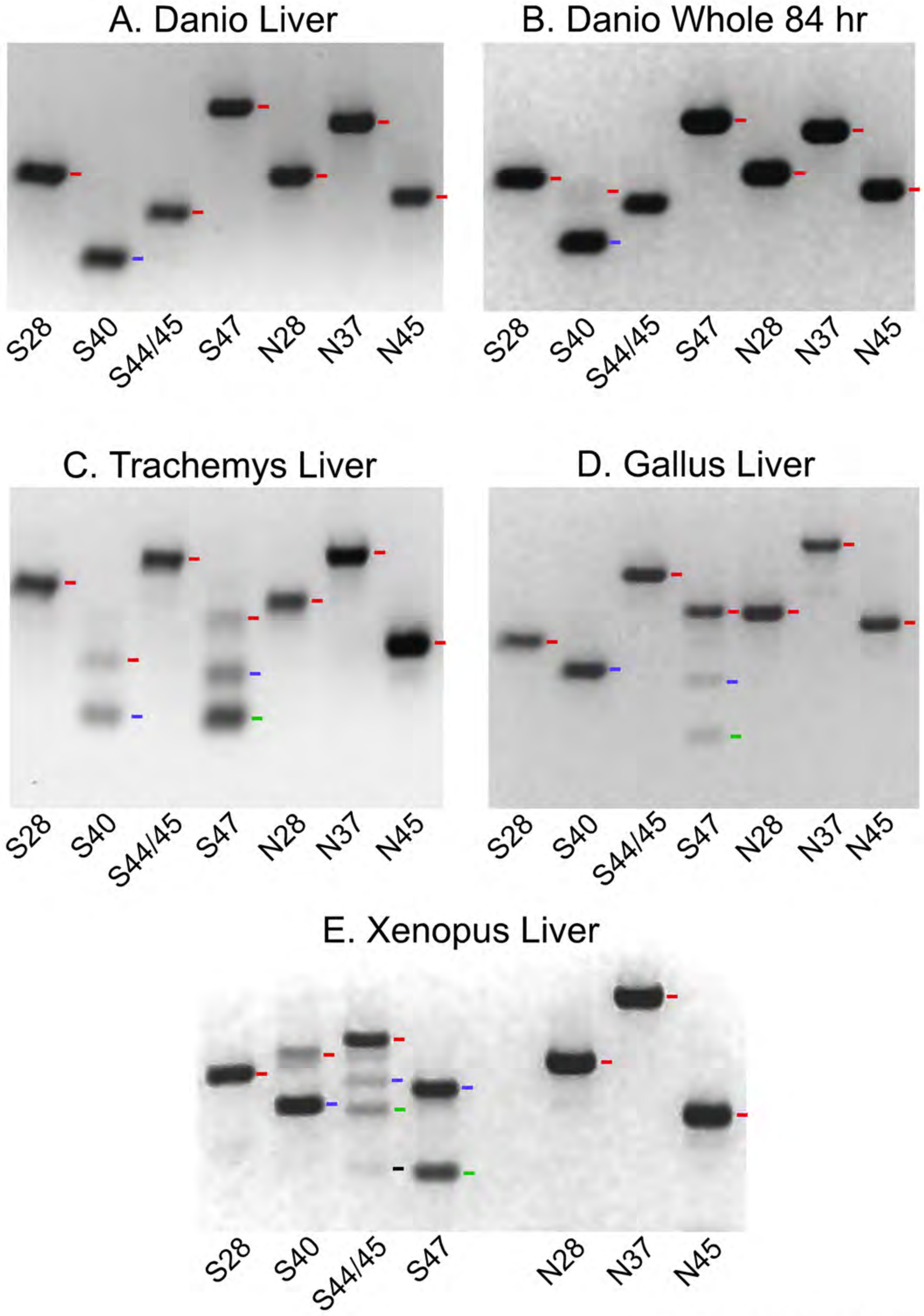

Supplemental Figure S1

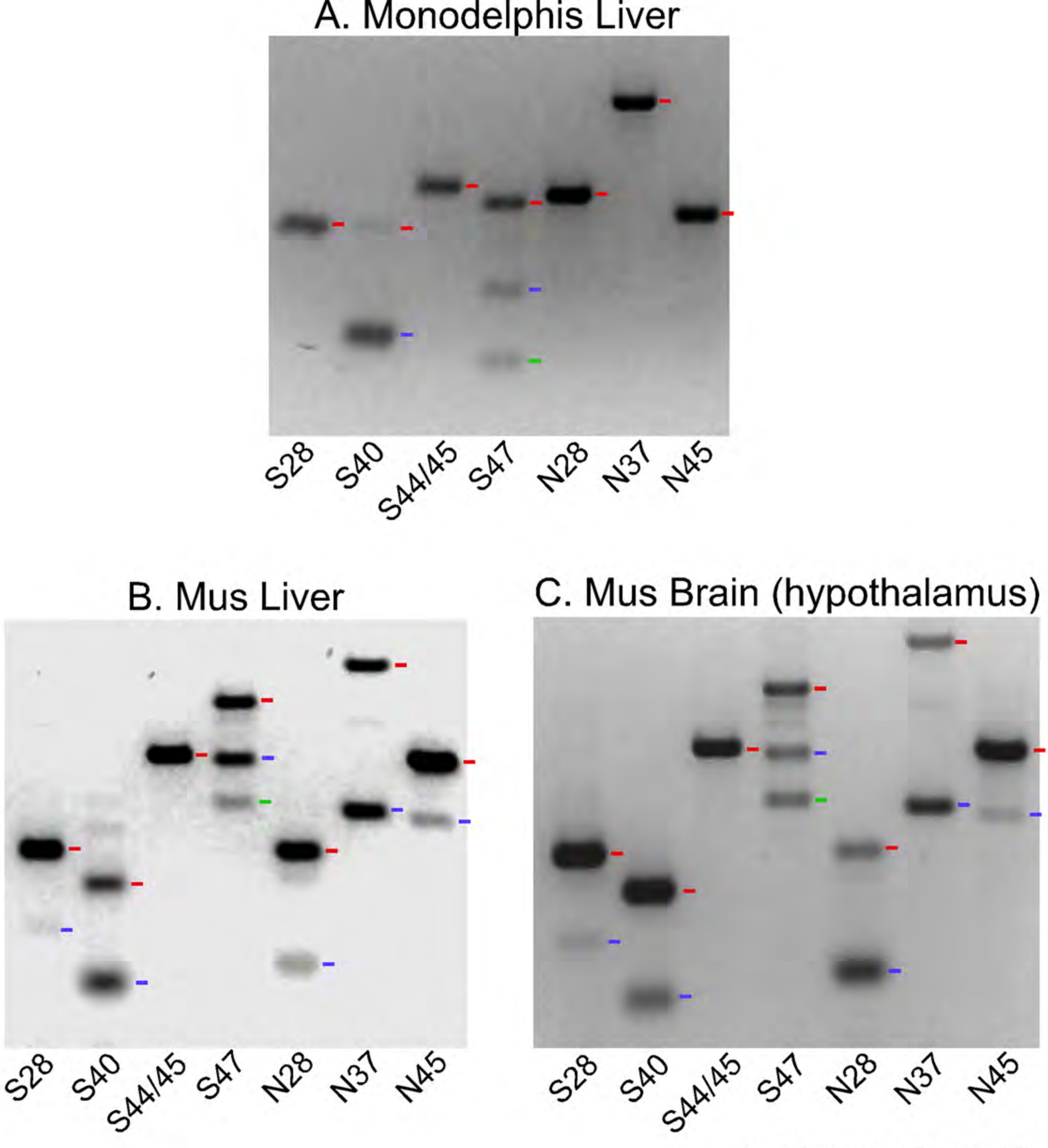

Supplemental Figure S2

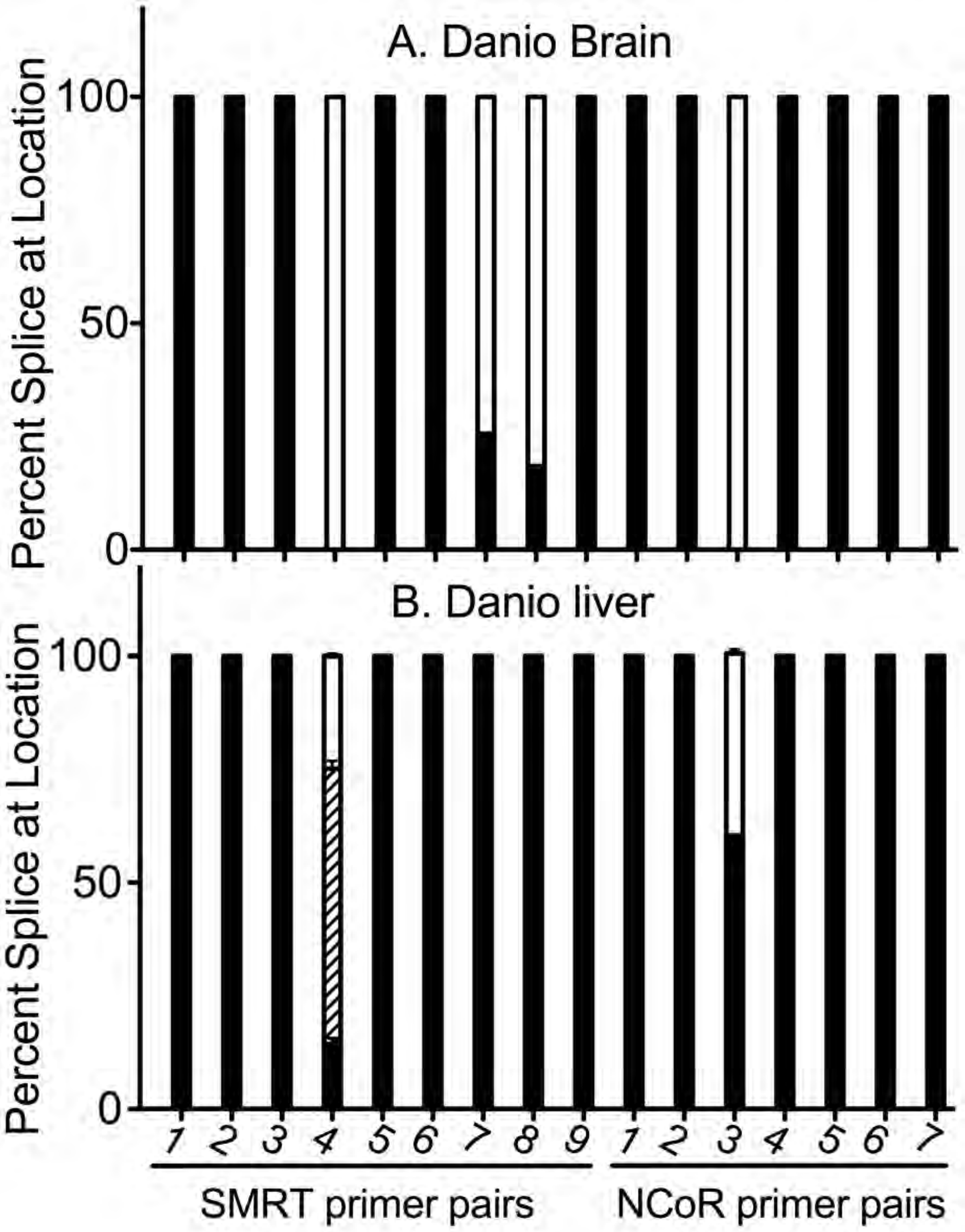

Supplemental Figure S3

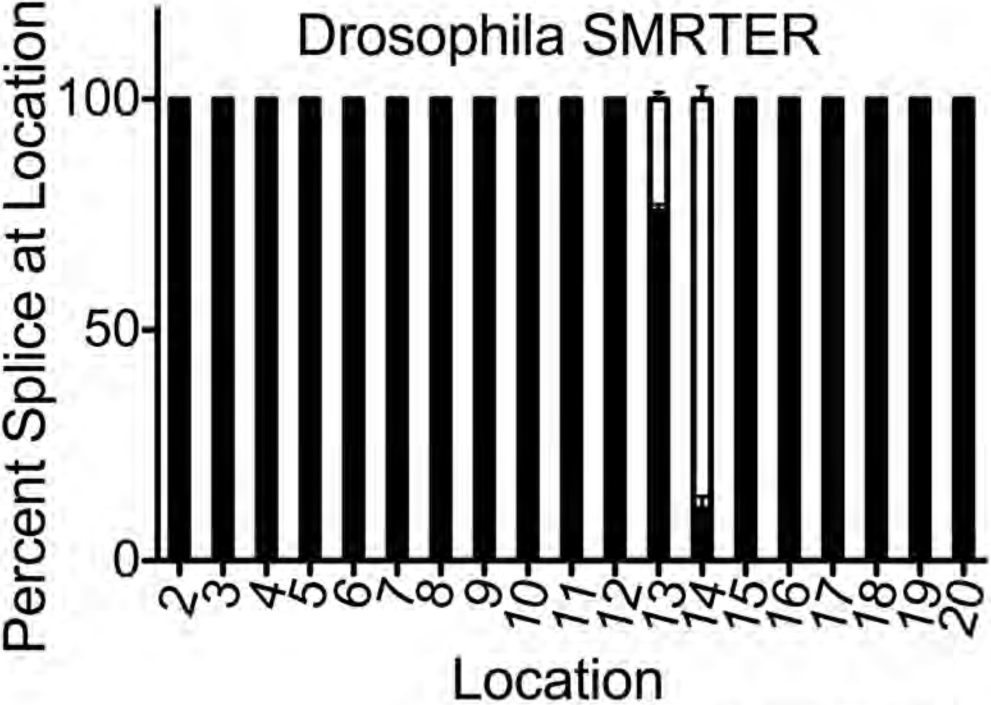

Supplemental Figure S4

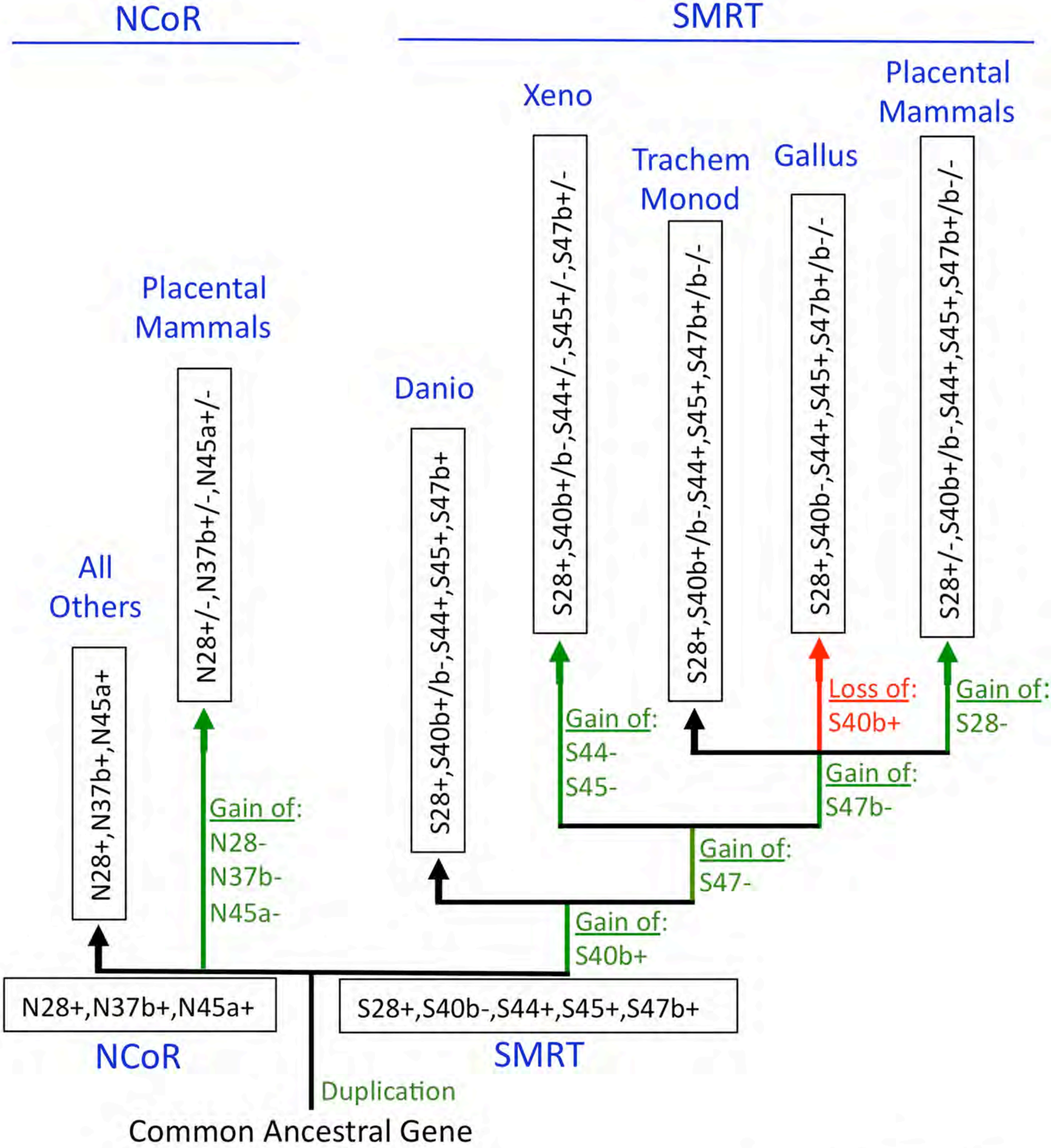

Supplemental Figure S5
